# Numerical simulation of patient-specific endovascular stenting and coiling for intracranial aneurysm surgical planning

**DOI:** 10.1186/s12967-018-1573-9

**Published:** 2018-07-21

**Authors:** Xiaochang Leng, Yang Wang, Jing Xu, Yeqing Jiang, Xiaolong Zhang, Jianping Xiang

**Affiliations:** 1 ArteryFlow Technology Co., Ltd, 459 Qianmo Road, Suite C1-501, Binjiang District, Hangzhou, 310000 Zhejiang Province China; 2 Department of Neurosurgery, The First Affiliated Hospital of Nanchang University, Nanchang University, Nanchang, China; 3 Department of Neurosurgery, The Second Affiliated Hospital of Zhejiang University, Zhejiang University, Hangzhou, China; 4 Department of Radiology, Huashan Hospital Affiliated to Fudan University, No. 12 Wulumuqi Zhong Road, Shanghai, 200040 China

**Keywords:** Stent, Coil, Aneurysm, Nitinol, FEM, CFD

## Abstract

**Background:**

In this study, we develop reliable and practical virtual coiling and stenting methods for intracranial aneurysm surgical planning. Since the purpose of deploying coils and stents is to provide device geometries for subsequent accurate post-treatment computational fluid dynamics analysis, we do not need to accurately capture all the details such as the stress and force distribution for the devices and vessel walls. Our philosophy for developing these methods is to balance accuracy and practicality.

**Methods:**

We consider the mechanical properties of the devices and recapitulate the clinical practice using a finite element method (FEM) approach. At the same time, we apply some simplifications for FEM modeling to make our methods efficient. For the virtual coiling, the coils are modeled as 3D Euler–Bernoulli beam elements, which is computationally efficient and provides good geometry representation. During the stent deployment process, the stent–catheter system is transformed according to the centerline of the parent vessel since the final configuration of the stent is not dependent of the deployment history. The aneurysm and vessel walls are assumed to be rigid and are fully constrained during the simulation. All stent–catheter system and coil–catheter system are prepared and packaged as a library which contains all types of stents, coils and catheters, which improves the efficiency of surgical planning process.

**Results:**

The stent was delivered to the suitable position during the clinical treatment, achieving good expansion and apposition of the stent to the arterial wall. The coil was deployed into the aneurysm sac and deformed to different shapes because of the stored strain energy during coil package process and the direction of the microcatheter.

**Conclusions:**

The method which we develop here could become surgical planning for intracranial aneurysm treatment in the clinical workflow.

## Background

Intracranial aneurysms (IA) affect up to 5% of the US population [1]. Rupture of IAs leads to subarachnoid hemorrhage, the most severe form of stroke with high rates of mortality (> 50% at 30 days) and disability (> 50% permanent disability among survivors) [2]. The traditional IA treatment entails clipping, an open-skull, maximally invasive surgery with significant morbidity and mortality. Since early 1990s, endovascular intervention of coil embolization is widely used as a minimally invasive alternative that has revolutionized IA treatment [3, 4]. Coil embolization obliterates an IA by filling the sac with platinum coils to reduce aneurysmal inflow and induce aneurysmal thrombosis. For wide-necked IAs (defined as having a neck of ≥ 4 mm or a dome-to-neck ratio of < 2), neurovascular stents are often deployed across the orifice of aneurysms in the parent vessel, typically a high-porosity neuro-stent is deployed across the aneurysm to reduce coil herniation [5]. This treatment strategy is called stent-assisted coiling. Approximately one-third of IAs use a stent to assist coiling. Though increasing reports of successful endovascular intervention, many events of failure to occlude of IAs [6] and permanent neurological procedure-related complications [7] occur.

Moreover, prospective randomized multicenter trials comparing clipping with coiling in both ruptured and unruptured IAs have demonstrated better outcomes with endovascular therapy [4, 8, 9]. However, the major drawback for endovascular treatment remains high recanalization (recurrence) rates (30%) [10–12] and the need for retreatment in coiled IAs [4]. Patients experiencing these negative outcomes are subjected to increased risk of IA rupture and complications from retreatment and generally have fewer treatment options available. Unfortunately, there is no way for clinicians to predict outcome of coiling intervention [9].

In this study, we develop reliable and practical methods for virtual coiling and stenting. As the purpose of deployment of coils and stents is to provide the geometries for accurate post-treatment computational fluid dynamics (CFD) analysis, methods of this study do not require capturing all the details such as the stress and force distribution for the devices and vessel walls. Our philosophy for developing these methods is to balance accuracy and practicality. In this study we develop reliable and practical methods using simplified finite element method (FEM) for virtual coiling and stenting. We consider the mechanical properties of the devices and recapitulate the clinical practice using a FEM approach. At the same time, we apply some simplifications for FEM modeling to make our methods efficient. Once these two methods are developed, standard CFD procedure can be applied to simulate post-treatment hemodynamics in patient-specific IAs to investigate the association and build a statistical prediction model between the flow dynamics and clinical treatment outcomes using large number of treated cases by coiling and stenting in the future. This prediction model will be able to help assess different treatment strategies and choose the optimal treatment option.

## Methods

### Aneurysm model

**Fig. 1:**
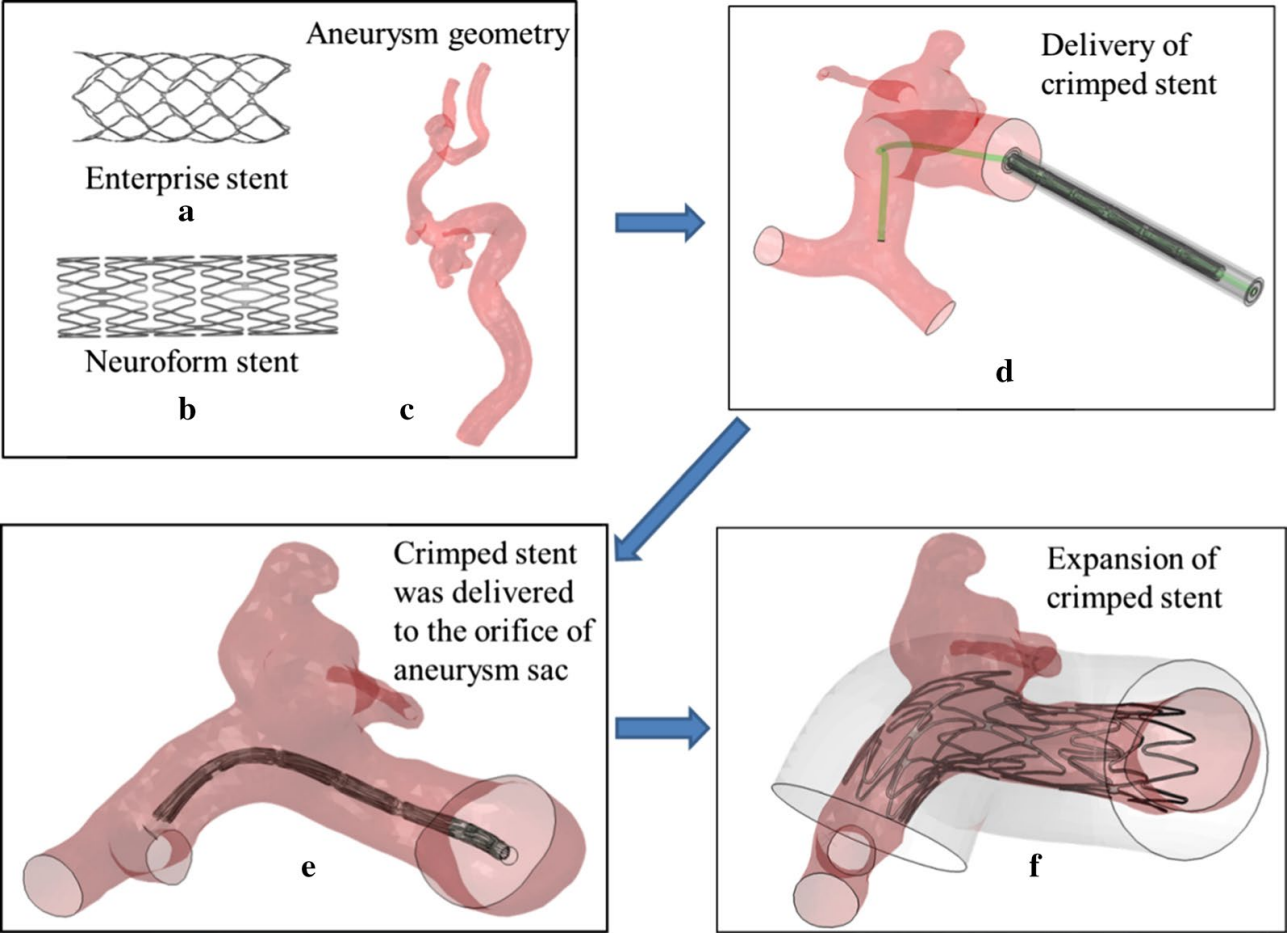
Flow chart for stent deployment: 3D geometry model of **a** Enterprise stent (4.5 mm × 14 mm) and **b** Neuroform stent (4.5 mm × 20 mm); **c** a patient-specific ICA aneurysm models used in this study; **d** delivery of stent near the aneurysm sac; **e** a crimped stent was delivered to the orifice of aneurysms in the parent vessel; **f** self-expansion of the stent and the configuration of a stent deployed in the lesion

Patients with intracranial aneurysms at Huashan Hospital, an affiliate of Fudan University between January 2017 and December 2017 were enrolled in this study. An internal carotid artery (ICA) aneurysm was used in this study for demonstrating our method. 3D rotational angiography images were obtained and 3D reconstruction in surface-triangulation format and isolation of the region of interest were performed using open source image tool-vmtk (http://www.vmtk.org). Then 3D segmented geometries were cleared in Geomagic tool (Geomagic Inc., Morrisville, North Carolina) and ready for FEM analysis, CFD meshing and simulation. Part of arterial wall with aneurysm sac was isolated from its parent vessel in Geomagic for the insertion of a microcatheter for coil deployment. The volume of this aneurysm was measured to be 116.64 mm^3^ as shown in Fig. 1.

### Stent and coil models

Neuroform stent and Enterprise stent are two most common types of stents used to assist coiling of the intervention treatment for intracranial aneurysms. The geometrical models were generated using SolidWorks (as shown in Fig. 1a, b) and transformed into finite element analysis (FEA) code ABAQUS for analysis.

According to previous work about a 3D parametric equation [13] for generation of a coil, a centerline of the coil was generated in MATLAB (MathWorks, Natwick, MA). There were two types of coils, one is the helical coil and another is the frame coil. For the efficiency of FEA process, the shape of the coil was simplified by using a centerline. The 3D shape of coils was assumed to be a continuous cylindrical structure [13, 14] and swept to 3D configuration when the coils were deployed in the aneurysm sac.

### Library for packaged coils and crimped stents

Coils were pulled into microcatheter, with a length equal to the length of coil, and the stents were compressed by a cylinder to a diameter equal or less than the microcatheter. These packed coils and crimped stents with different dimensions were prepared for the virtual stenting and coiling. Building this library for packaged coils and crimped stents will decrease the time for simulating coil and stent deployment.

### Structural simulations of stent deployment

#### Material property

Nitinol is a common material that used for the medical industry to construct self-expandable stents for clinical treatment of intracranial aneurysm and atherosclerosis [15]. The material properties of nitinol were obtained from previous work [16–18], as shown in the Table 1.

**Table 1:** SMA material properties for the Auricchio/Taylor superelasticity model [17, 18]

Thermoelastic properties			
*E* ^*A*^	*E* ^*M*^	*v* ^*A*^	*v* ^*M*^
70 GPa	70 GPa	0.33	0.33

Phase diagram properties							
$\sigma^{{M_{s} }}$	$\sigma_{C}^{{M_{s} }}$	$\sigma^{{M_{f} }}$	$\sigma^{{A_{s} }}$	$\sigma^{{A_{f} }}$	*C* ^*A*^	*C* ^*M*^	*T*_0_
448 MPa	448 MPa	562 MPa	257 MPa	221 MPa	9.21 MPa/K	6.31 MPa/K	350 K

Transformation strain properties	
*H*	*H*_*V*_
4.7%	4.7%

#### Workflow for stent deployment modeling

The FEA-based workflow for stent deployment modeling was completed in ABAQUS/Explicit v6.14 (SIMULIA, Providence, RI). The workflow consists of three steps: delivery, pre-deployment and deployment.

Simulations of the stent deployment and coil delivery process were implemented via the general-purpose FEA software ABAQUS 6.14 in Abaqus/Explicit mode [19]. Simulations of the stent crimping, delivery and release processes were performed, in which the nitinol superelasticity material model were used. The virtual deployment of stent in terms of the delivery process through a path created with central points of the cross-sections of the blood vessel was implemented, which simulated the process of delivery of a stent during clinical treatment.

The crimped stent was assembled in a solid tube in the global coordinate system and transformed to the aneurysm orifice. This process was used to make the deformation of stent according to the axes of the blood vessel across the aneurysm orifice (as shown in Fig. 1d, e). At last, the microcatheter was applied with displacement load along the radius direction; the crimped stent was expanded itself in order to simulation the self-expansion procedure during clinical treatment (as shown in Fig. 1f).

In simulations, a “general contact” algorithm in ABAQUS was utilized for the complex interactions involved in stent deployment and a friction coefficient value of 0.15 was used for the current study.

### Stent deployment modeling with deformable arterial wall

For stent deployment modeling process, comparison of rigid wall between deformable arterial wall is essential for CFD study of virtual clinical planning. Thus, the cerebral arterial wall was modeled as Holzapfel–Gasser–Ogden (HGO) [20] material model, which was used to capture the bulk arterial material behavior. Due to the limited HGO parameter values for cerebral artery, a set of parameter values from carotid arterial wall or abdominal arterial wall were adopted, which the parameters were chosen as μ = 3.82e−3 MPa, k_1_ = 0.996 MPa, k_2_ = 524, κ = 0.226 and the collagen fibers angle is 49.9 [20–23]. The aneurysm vessel wall was model as membrane element with a thickness of 0.3 mm [24].

### Structural simulations of coil deployment

**Fig. 2:**
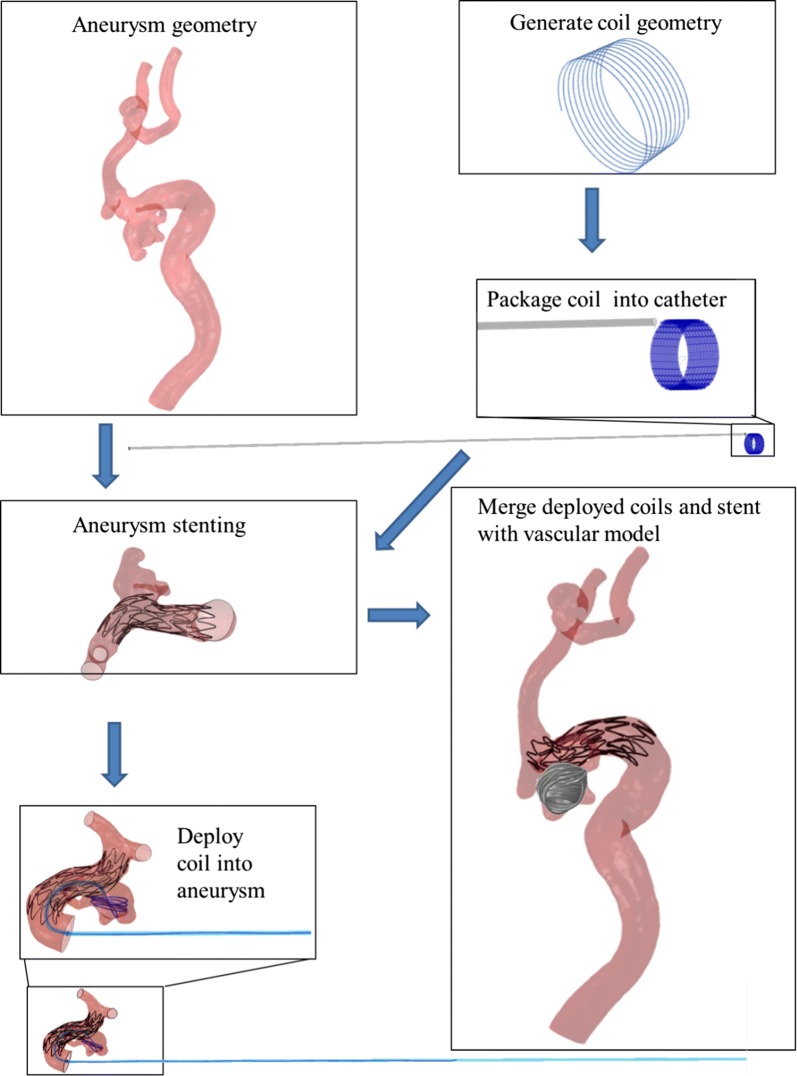
Flow chart for coil deployment, including aneurysm stenting, coil package, coil deployment and merging deployed coils with vascular model

The flow chart for coil deployment is shown in Fig. 2. The coil centerline was imported into the FEM code ABAQUS and meshed with an Euler–Bernoulli beam element type. In order to diminish coil-to-coil and coil-to-sac surface penetrations during deployment, the diameters of the coils were set equal to 1.5 times of the real size [13, 14], helical coil with 0.45 mm. A linearly-elastic constitutive mechanical model with isotropic material properties was adopted, with a Young’s modulus of 7500 MPa, Poisson’s ratio of 0.39 and density of 0.0213 g/mm^3^ [13].

After generating different types of coils, the coils were packaged into a virtual microcatheter, which was created as a 3D rigid shell extrusion, with a length equal to the length of coil and diameter of 0.6 mm. The other part of microcatheter was created along the center line and across the center of one cell of stent in order to mimic the surgical process after the microcatheter delivery to the sac. One end of coil was fixed to the proximal of microcatheter with a spring element in order to constrain the movement of the coil. The other end of coil was pulled forward into the microcatheter by applying a displacement of the length of microcatheter. The rest part of coil is under stress-free condition. During this process, the catheter and sac were set as a rigid body in ABAQUS without any movement. General contact algorithm in Abaqus/Explicit was implemented for the interaction between coil, catheter, with frictionless for the tangential direction and a “hard”-contact for the normal direction behavior. The next step after “package of coil into catheter” is “deploy coil into aneurysm”. At the beginning, the catheter and aneurysm sac were set as a rigid body fully constrained. By applying displacement on the proximal end of the coil, the coil was push forward into the sac with stress-free condition. A general contact algorithm as the previous step was defined for the interaction between coil and catheter and coil between sac, with friction coefficients of 0.2 for the tangential friction behavior [13, 14] and a “hard”-contact for the normal direction behavior. The internal strain energy accumulated for the coil during the coil-package process was release after the coil was pushed into the sac, and the coil spring back to its previous shape within the aneurysm.

After all the coils have been deployed into the aneurysm sac, the coils were sweep to 3D solid model by using Abaqus/CAE with the real diameter of the device. To the end, the 3D representation of the coil can be used for the CFD analysis with a surface-based boundary condition.

## Results

### Library for packaged coils and crimped stents

**Fig. 3:**
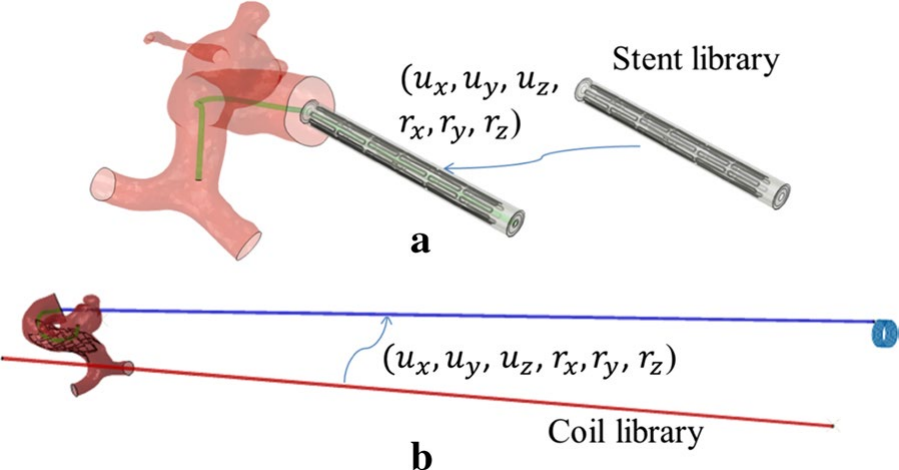
**a** Stent–catheter and **b** coil–catheter packages from the library were transformed and ready for deploying to the aneurysm sac

The library for packaged coils and crimpled stents are shown in Fig. 3a, b, which can be transformed to the position for the deployment. The packaged coils and crimped stents can be prepared at the beginning of the simulation which can be very efficient for the virtual treatment.

### Virtual stenting results

**Fig. 4:**
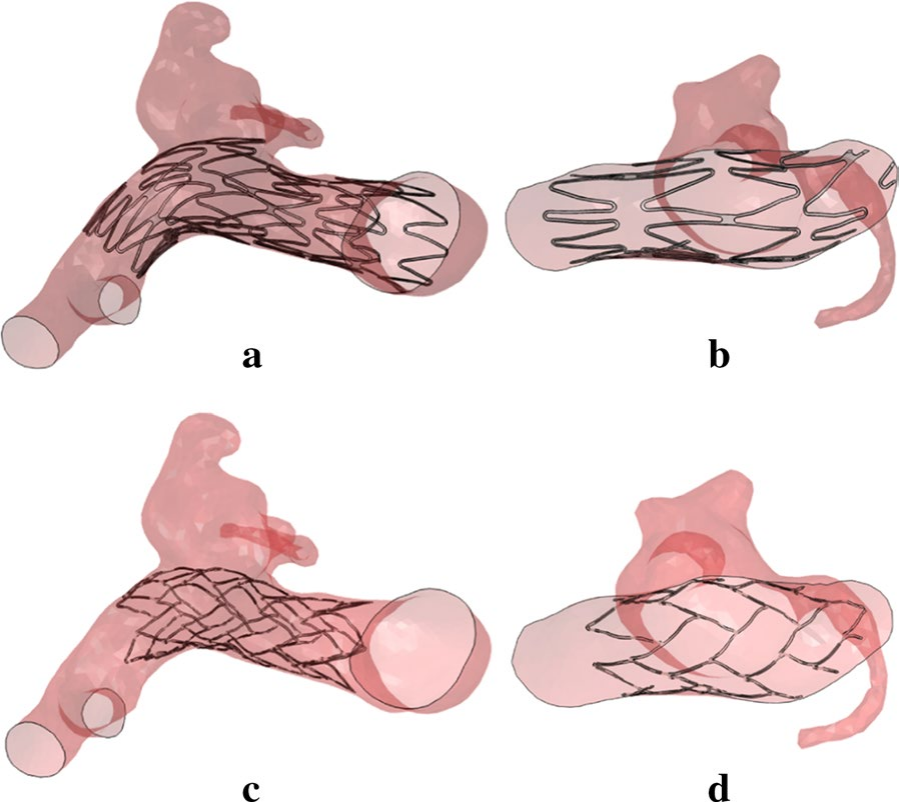
**a**, **b** Neuroform stent and **c**, **d** Enterprise stent deployment results in the same aneurysm patient

According to the streamlined virtual stenting workflow, two types of stents were deployed in the same aneurysm; the configurations of stents are shown in Fig. 4. The cells of stent were distributed across the orifice of giant, wide-necked and fusiform aneurysms in the parent vessel in order to prohibit the coils out of the aneurysm sac. The stent was delivered to the suitable position during in the clinical treatment, achieving good expansion and apposition of the stent to the arterial wall.

### Result of stent deployment modeling with deformable arterial wall

**Fig. 5:**
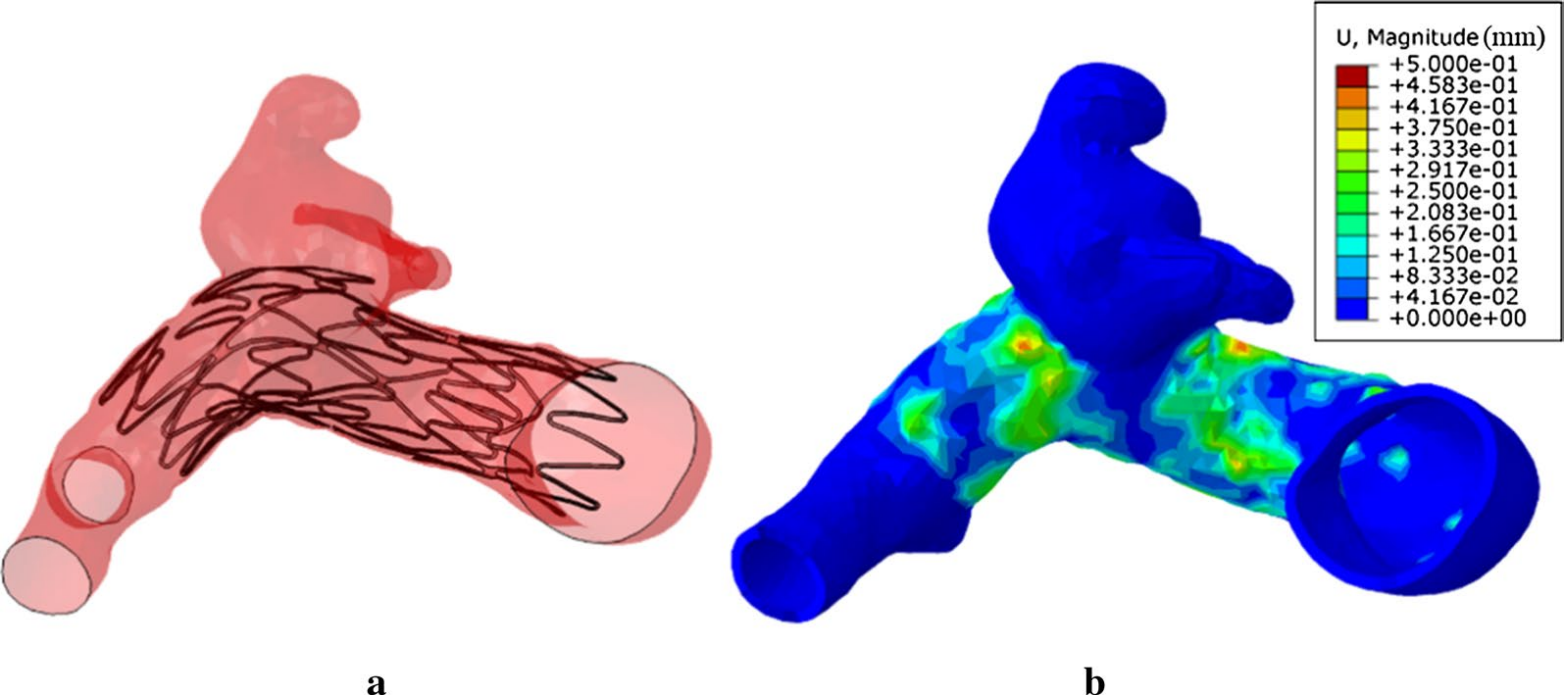
**a** Self-expansion of the stent and the configuration of a stent deployed near the sac when it was set with deformable arterial wall. **b** The contour of the magnitude of displacement for arterial wall

In order to find out the difference between the configurations of the aneurysm vessel wall when it is modeled as rigid wall and deformable wall, a HGO model was adopted to capture the aneurysm–vessel wall deformation. The stent–wall configuration is shown in Fig. 5a, which revealed that the shape of the arterial wall has small change that the wall expanded radially to about 0–0.45 mm, especially the area contacted by the head of cell of stent, as shown in Fig. 5b. However, overall the deformation of aneurysm and vessel was minimal after the stent devices were deployed as demonstrated in this study.

### Virtual coiling results

**Fig. 6:**
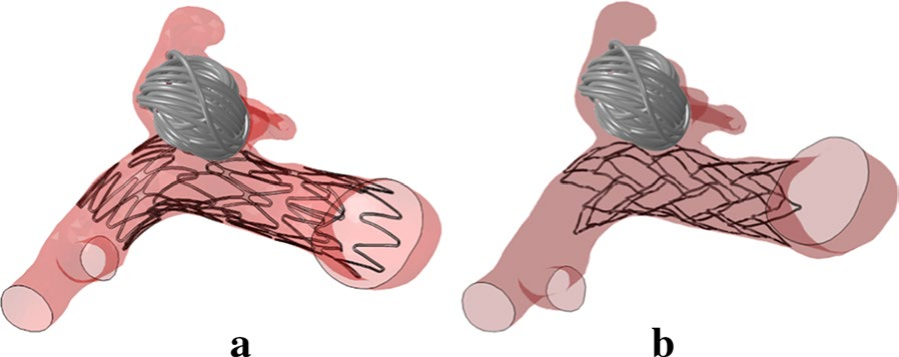
Coils deployment in the same aneurysms with two different types of stenting: **a** Neuroform stent; **b** Enterprise stent

To illustrate the workflow of coil deployment, the final configurations of the coils are shown in Fig. 6. The coil was deployed into the aneurysm sac and deformed to different shapes because of the stored strain energy during coil package process and the direction of the microcatheter.

## Discussion

In this paper, we developed a reliable and efficient surgical planning procedure for intracranial aneurysms. For virtual surgical planning of stent and coil deployment, though some simplifications were applied, the main feature of this process were captured, including the final configurations and positions of the stent and coil, and these results were sufficient for providing the geometries of coils and stents for subsequent accurate post-treatment CFD analysis. The steps to link these modeling techniques with ultimate surgical outcome includes: (1) develop modeling techniques from current study; (2) validate these modeling techniques; (3) once these two methods are developed and validated, standard CFD procedure can be applied to simulate post-treatment hemodynamics in large number of patient-specific IAs; (4) investigate the association and build a statistical prediction model between the flow dynamics and clinical treatment outcomes using large number of treated cases by coiling and stenting. This prediction model will be able to help assess different treatment strategies and choose the optimal treatment option.

Currently, both porous media methods [25, 26] and fast virtual methods such as dynamic path planning for coiling [27] and simplex mesh expansion for stenting [28] are available. They are fast; however, their accuracy is greatly compromised. By representing coils [25, 29] and stents [26] as homogeneous porous media, they do not sufficiently capture specific flow interventions. Furthermore, it is tedious to determine patient-specific coefficients for porous media models. The existing expansion-based fast virtual stenting includes the collision detection process [28], which is inherently unstable, while the existing dynamic path planning method for fast virtual coiling [27], based on an artificial potential field, is not realistic as it does not take into account the coil pre-shape. The FEM has been applied to virtually deploy stents and coils [30, 31]. FEM-based techniques are accurate owing to explicit mechanical representation of device deployment. However, as they were used to capture all the details of the mechanical properties and behaviors of the devices and vessels, they are computationally expensive and time-consuming, and thus not practical. For example, a FEM-based HiFiVS technique for flow diverter (FD) deployment takes 100 h for single stent deployment [30]. Coils alter aneurysmal hemodynamics by reducing aneurysmal inflow, which initiates subsequent thrombosis within the aneurysm, leading to occlusion of the aneurysm and its eventual exclusion from the blood flow circulation. However, flow impingement acting on coil mass or on the aneurysm wall is believed to be responsible for the coil recanalization of coil-treated IAs. Therefore, knowledge about the impact of coils or stent-assisted coils on hemodynamics is critical for predicting coiling treatment outcomes. Image-based CFD analysis can provide detailed information on post-treatment hemodynamics, but it requires realistic representation of coils and stents in deployed states. This presents challenges to the numerical simulation of coil and stent implantation, as previous methods do not efficiently capture realistic coil and stent deployment.

This study focuses on generating accurate deployed coil and stent geometries for subsequent post-treatment flow simulation, taking no account of capturing all the details, i.e., the stress or force distribution. The simplifications used in this paper include: (1) coils are modeled under the assumptions of 3D Euler–Bernoulli beam elements as coils are slender in shape with the length dimension that is much larger than the diameter. It is computationally efficient and provides good geometry representation. The complex-shaped coils (free-stress status without strain energy) will firstly be “pulled” into the catheter to package them (with strain energy) and then “pushed” into the aneurysm sac via a catheter. Though the 3D configuration of coils may be different from that of the clinical treatment, the packing density will be almost the same. Morales et al. have demonstrated that the simulated flow field was independent of coil deployment, when packing density is greater than 22% [37]. (2) During the stent deployment process, we directly transform the stent–catheter system according to the centerline of the parent vessel since the final configuration of the stent is not as dependent on the deployment history as the FD. (3) The aneurysm and vessel are assumed to be rigid and are fully constrained during the simulation, because the deformation of aneurysm and vessel is minimal after the stent devices were deployed as demonstrated in this study (Fig. 5b). Neuro-stents to assist coiling are self-expandable stents with small radial force, thus rigid wall assumption is sufficient. This is different from the balloon-expandable stent deployment for stenosis-like coronary artery disease, where the contact stress applied to the stenosis from the opening stent, and thus rigid wall assumption can’t capture the deformable arterial wall impacted by stent. Moreover, the angle of aneurysm vessel may change from a sharp-angle configuration to a straight line shape when the radial force applied to the vessel wall from the expansion of stent. This change will have a certain effect to the overall shear stress, compression stress and velocity of the blood flow when the CFD analysis is conducted [32].

Typically during FEM simulation procedures, packaging a coil into the catheter take around 1 h and crimping a stent could take up to 5 h. Building a library of packaged coils and crimped stents with different dimensions will significantly reduce the simulation time for future cases. After building the library, for virtual coiling we only need to simulate pushing the coil out of the catheter to deploy it into the aneurysm sac; while for virtual stenting, we will only need to transform the system and retract the catheter to release the stent. We expect both coiling and stenting simulation procedures to take around 1 h based on the library. Compared with the previous HiFiVS technique of FD deployment, which takes around 100 h [30], our proposed methods are very efficient and practical. Even compared with current fast virtual intervention methods, which typically take minutes, our current methods are quite reasonable. Since post-treatment CFD simulations take up to hours for flow simulations even on computational clusters regardless of the device deployment methods used, the additional time needed for FEM-based over fast virtual deployment methods is insignificant. However, our simplified FEM-based coiling and stenting methods intrinsically capture the mechanical features and promise to more accurately recapitulate the device development.

## Limitation

There are several limitations of this study. First, we did not conduct the validation of stent and coil deployment results in vitro and in vivo. We will conduct the validation in the future by both in vitro and in vivo data as follows:

1. Validate the flow field based on the deployed geometry using in vitro data:
  We will choose 12 patient-specific aneurysm 3D angiographic images for the algorithm validation based on different aneurysm types and locations. Two-thirds of the patients (8 aneurysms) have narrow-neck aneurysms treated by coiling alone, while the rest one-third of the patients (4 aneurysms) have wide-neck aneurysms treated by stent-assisted coiling to match the ratio of clinical coiling treatment strategies. These 3D IA images will be segmented to obtain the 3D geometries with STL files. From the STL files, we will build aneurysm phantoms made of elastomer as described [33] and deploy coil and stent devices as done in the real patients. We will connect the phantoms to a flow loop mimicking cerebral blood flow and use stereoscopic Particle Image Velocimetry (PIV) to measure the post-treatment 3D pulsatile aneurysmal flow fields (at peak and end of diastole) for flow validation. Specifically, we will use PIV to measure the 3D velocity field only at the IA neck plane as the flow field inside the sac could not be measured by PIV due to the reflection of densely packaged coils. Measured PIV images will be automatically uploaded into the PIV post-processing system to generate in-plane velocity vectors, magnitude contour and out-of-plane velocity vectors. Flow patterns at the IA neck plane will be qualitatively compared for each case between PIV measurement and CFD simulation.
2. Validate the flow field based on the deployed geometry using in vivo data:
  For in vivo validation, we will generate virtual angiograms from the CFD results of these 12 cases by simulating scalar transport and contrast density projection [34, 35], and compare them with clinical angiograms. Comparison will be done both qualitatively (flow patterns including jets, recirculation zones) and quantitatively (contrast residence time).

We expect that in vitro and in vivo testing will validate the accuracy of our simplified FEM-based virtual coiling and stenting methods in calculating hemodynamic parameters of patient-specific IAs. Especially for the primary coiling treatment (current study scope), coil packing density is always around 27–28% clinically [36]. Frangi et al. have demonstrated that the simulated flow field was independent of coil deployment, when packing density is greater than 22% [37].

Second, the material parameter values chosen for capturing deformable cerebral arterial wall was based on the values from carotid or abdominal arteries, which may be not appropriate for the cerebral artery. We will obtain the HGO parameter values from tensile tests of human cerebral arterial vessels from autopsy study by cooperating with hospitals in the future. We will then do the simulation by replacing the material parameter values from experiments. Third, we simulated the stent and coil deployment process to only one patient-specific aneurysm, which is not sufficient to cover the different shapes of aneurysms.

## Conclusion

In this study, two types of surgical planning simulation were performed, the stent deployment and coiling. A simplified modeling approach to simulate and understand these processes were developed. In particular, the use of 3D Euler–Bernoulli beam elements for modeling coils and transformation according to the centerline of the parent vessel for the delivery of stent–catheter system were carried out. The aneurysm and vessel are assumed to be rigid and are fully constrained during the simulation, and all the stent–catheter systems and coil–catheter systems were prepared and packaged as a library which contained all types of stents and coils. It is computationally efficient and provides good geometry representation, which can provide the geometries for subsequent accurate post-treatment CFD analysis.

## Abbreviations

CFD: computational fluid dynamics
FEM: finite element method
IA: intracranial aneurysms
ICA: internal carotid artery
HGO: Holzapfel–Gasser–Ogden
FD: flow diverter
